# Livestock feed resources in the West African Sahel

**DOI:** 10.1002/agj2.20955

**Published:** 2021-12-29

**Authors:** Tunde Amole, Ayantunde Augustine, Mulubrhan Balehegn, Adegbola T. Adesogoan

**Affiliations:** ^1^ Feed and Forages International Livestock Research Institute Ibadan Nigeria; ^2^ International Livestock Research Institute Ouagadougou Burkina Faso; ^3^ Feed the Future Innovation Lab for Livestock Systems, Mekelle, Tigray; ^4^ Dep. of Animal, Rangeland and Wildlife Sciences, Mekelle Univ., Mekelle, Tigray

## Abstract

Limited supply of quality feed is the most important factor limiting livestock productivity in many sub‐Saharan African (SSA) countries. Having a systematic inventory of available feed resources, identifying main challenges and potentials for improvement is the first step towards designing development strategies to improve feed quality and quantity. The objective of this study was to review the available feed resources and their quality in West African Sahel across different agro‐ecological zones and to identify the research gaps and strategies to improve feed resource availability. The West African Sahelian zone is home to 135 million people who herd 173 million head of ruminant livestock. The main feed resources for grazing ruminants are pastures and crop residues; commercially formulated feeds are increasingly being used in poultry and pig production, particularly in peri‐urban areas. Feed resources for livestock are diverse and vary markedly across agro‐ecological zones in the West African Sahel and across seasons in terms of type, quantity, and quality. Given that crop residues are among the most important feed resources, there is need to invest in promoting adoption of proven methods for improving their quality and preserving it. Given poorly developed feed markets in the Sahelian rural areas and cities, strengthening the feed value chain is critical for improving the feed resource base in West Africa. Additional critically important needs are to increase awareness about the importance of feed quality, to create quality‐based feed marketing systems, and to appreciate and enhance women's roles in feed production.

## Core Ideas

- Productivity of livestock in West Africa Sahel is constrained by limited supply of quality feed.
- Feed quality is low, and availability varies seasonally, reducing livestock productivity.
- Rangeland, crop residues, and agro‐industrial by‐products are important feed resources in Sahel.
- Improving nutritive quality of crop residues, fodder preservation, and better feed marketing are needed.
- Identifying available feed resources is necessary for designing interventions for improvement.

**Abbreviations**
AIBP: agro‐industrial by‐product
CP: crude protein
DM: dry matter
IP: innovation platform
LW: live weight
OM: organic matter
SSA: sub‐Saharan Africa.

## 1 INTRODUCTION

Livestock .husbandry is critical for many smallholder farmers in the West African Sahel, often contributing to multiple livelihood objectives and offering pathways out of poverty (Randolph et al., 2007). Livestock provide traction to cultivate fields, manure to improve soil fertility and nutritious food products for human consumption, and income generation (Sere et al., 2008). In the West African Sahel, livestock production systems are diverse, ranging from extensive pastoral systems to mixed crop and livestock systems. Over a decade ago, the report of the Sahel and West Africa Club hosted at the Organization for Economic Cooperation and Development of the Economic Community of West African States (Kamuanga et al., 2008) indicated that the livestock sector provided between 8 and 15% of overall gross domestic product (GDP), 44% of agricultural GDP, and nearly 50% of the workforce engaged in the sector in West Africa.

Globally, ruminant nutrition depends largely on naturally growing pastures, which fluctuate both in quantity and quality within the year (Wanapat, 2000). In the Sahel, in addition to the rangelands, many livestock farmers depend on crop residues, which are an important feed resource during the dry season (Teferedegne, 2000). These crop residues are becoming a dominant feed resource as rangelands are being converted into crop fields in the Sahel (Herrmann et al., 2020). The proportion of crop residues in the animal diet is related to annual rainfall, the intensity of cropping, and the available forage during the dry season (Ayantunde et al., 2000). Associated with the seasonal fluctuation in quantity and quality of feed resources is poor nutrition of the animals, which is a major constraint to livestock production in West African Sahel. Hence the cycle of weight gain in the wet season and weight loss in the dry season is a common feature of livestock body weight development in the Sahel. For instance, in a study in Niger, following closely the pattern of forage availability, local breed cattle gained 80–100 kg during about 6 mo (July–December) followed by a loss of 60–80 kg during about 6 mo (January–June) with a net weight gain of about 20 kg per year, whereas small ruminants showed much shorter periods of loss and relatively higher weight increase per year (Fernández‐Rivera et al., 2005). There is no standard duration for raising the animals to slaughter as sale of male animals is largely driven by household cash needs. For example, a male calf of 1‐yr old can be sold if the owner has cash needs. From the survey in southwestern Niger, more than 95% of cattle, sheep, and goats sold are males as the females are always retained for herd productivity. Cows are only sold when they are old (often more than 9 yr) and are no more producing. (Ayantunde et al., 2007; Wilson, 1986). The average body weight of matured cattle in the Sahel varies but it generally ranges from 220 to 300 kg (Wilson, 1986). It is difficult to estimate conception rate (conception at first service) or pregnancy rate (percentage pregnant per service) for smallholder production systems in the Sahel, as the systems are extensive, and mating is not often monitored. The bull used for mating heifer or cow may be selected especially in pastoral herd to breed for certain desirable traits, but the actual mating is often not monitored (Ayantunde et al., 2007). For cattle breeds in the Sahel, the calving rate is about 60% and parturition rate for cow of 4 yr and older is between 0.54 and 0.71 (Wilson, 1986).

By 2030, urban beef consumption is projected to grow by more than 361% in developing countries, while rural beef consumption will more than double (FAO, 2011). Such growth in consumption will largely drive associated growth in demand for feed. This creates a tremendous opportunity for increasing livestock productivity through feed supply, particularly as good quality feed is the most important constraint limiting livestock productivity globally (Phillip et al., 2009), and particularly in the Sahelian countries. The World Bank (2012) also predicted that changes in beef demand and supply in West Africa will likely result in increased sourcing of ruminant livestock feed from the market (13% in 2030 as compared to 2.5% in 2010) rather than from grazing. For the West African Sahel to be well positioned for responding to the present and future increase in the demand for livestock products, its feed resource base must be proportionally aligned with such increasing demand.

There have been several studies on evaluating feed resources for livestock production with the emphasis on identification of alternative feed sources to minimize production cost without reducing animal productivity in the long run (Amole & Ayantunde, 2016a, 2016b). Other studies have focused on an inventory of indigenous feed resources (Aduku, 2004; Bayala et al., 2014; Sanon et al., 2007) in the Sahel and the introduction of improved genotypes, especially as fodder banks (Bayala et al., 2014; Sanon & Kanwé, 2002). A comprehensive assessment of available feed resources; their problems; challenges including quality, availability, and other social challenges and opportunities for improvement, however, is not available. Identification and analysis of such issues is important for solving the problem of limited supply of quality feed in the Sahelian zone of Western Africa. The aim of this review is to synthesize previous studies on feed resources in West African Sahel, to identify gaps in knowledge, and strategies to increase the existing feed resource base. In addition, this review aims to generate baseline data to identify the potential crop residues within the major agro‐ecological zones of West Africa. Geographically, our review targets the West African Sahel, which covers Senegal, Mali, Burkina Faso, and Niger. We concentrated on Burkina Faso and Niger but also included relevant information from Senegal and Mali.

**Core Ideas**

- Productivity of livestock in West Africa Sahel is constrained by limited supply of quality feed.
- Feed quality is low, and availability varies seasonally, reducing livestock productivity.
- Rangeland, crop residues, and agro‐industrial by‐products are important feed resources in Sahel.
- Improving nutritive quality of crop residues, fodder preservation, and better feed marketing are needed.
- Identifying available feed resources is necessary for designing interventions for improvement.

## 2 METHODS

### 2.1 Study region

The region of focus for this review is the West African Sahel, which includes Burkina Faso, Mali, Mauritania, Niger, and Senegal. The Sahelian zone of West Africa is delineated by the 100‐mm isohyet in the North and 600‐mm isohyet in the South (Ayantunde, 1998). The Sahelian zone can be subdivided into three phyto‐geographical subzones namely the Sahara–Sahel between 100 and 200 mm, the “typical Sahel” from 200 to 400 mm, and the Sahelo‐–Sudanian zone between 400 and 600 mm. Basic information on land area, livestock population, and natural resources in the study region are presented in Table 1. In the West African Sahel, livestock form a key part of food security and livelihood strategies as they provide meat, milk, draught power, and manure for crop fields and fulfil various socio‐cultural functions. The main forms of livestock production system in the region are pastoralism and mixed crop–livestock systems. Cattle, goat, and sheep are the dominant livestock species in the Sahel. Peri‐urban livestock production is growing in importance in the major cities and towns of the West African Sahel, and this is driven by growth in demand for livestock products in peri‐urban areas (Ayantunde et al., 2018). The peri‐urban livestock system is characterized by small ruminant fattening, smallholder dairy production, and small commercial poultry production.

**TABLE 1:** Information on the land area, livestock population and natural resources in West Africa Sahelian countries

			Livestock population in 2018^b^			Natural resources in 2018^b^		
Country	Land area^a^	Percentage of Sahelian zone^a^	**Cattle**	**Goat**	**Sheep**	**Rangeland**	**Tree covered area**	**Shrub covered area**
	×1,000 km^2^	%	million heads			×1,000 ha		
Burkina Faso	274	7	9.84	15.64	10.44	31,314	2,182	3,776
Mali	1,220	40	11.76	25.22	18.27	65,503	5,437	10,122
Mauritania	1,025	39	1.92	7.47	11.02	28,776	311	1,188
Niger	1,267	50	14.36	17.41	12.75	41,054	1,037	3,135
Senegal	193	27	3.63	6.05	7.13	17,757	5,302	7,796

^a^Kamuanga et al. (2008).

^b^FAOSTAT (2021).

### 2.2 Literature search

**FIGURE 1:**
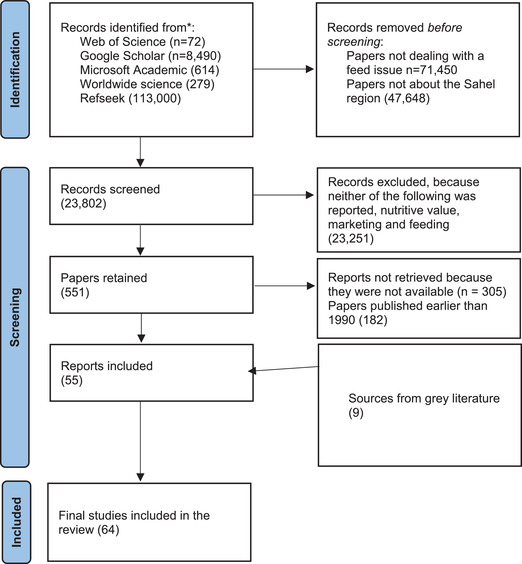
PRISMA diagram showing the total number of articles initially surveyed, the number included and excluded for this review

A search for relevant studies and publications was made using Web of Science, Google Scholar, Microsoft Academics, Refseek, and Worldwide Science Search databases. Various combinations of the following keywords were used: feed, livestock, Sahel or names of individual countries in the Sahel. The results were examined by reading the title and the abstracts to decide on whether to include a publication. Only studies that reported on feed resources in the Sahel area were included. The number of studies retrieved were 122,455 in the initial search and 98,653 were screened by removing those papers that dealt with nonfeed issues or with non‐Sahel regions. Of those retained for further screening, papers that reported either on nutritive value, marketing, or production aspects of feed resources in the Sahel (*n* = 551) were identified and included in the analysis, while the others were eliminated (*n* = 23,802). Subsequently, papers published before 1990 that reported on a feed resource for which there was a more recent information available were removed (*n* = 487). However, seven papers published earlier than 1990 were retained based on their high relevance. This process resulted in a final number of 55 papers that were included in the study. In addition to these journal papers, nine technical reports and gray literature papers that did not show up in the initial search were included, resulting in a total of 64 papers that were included in the study. Figure 1 provides the outline of the searching strategy used to identify papers that were included in the review.

## 3 RESULTS

### 3.1 Types and availability of feed resources in West African Sahel

**FIGURE 2:**
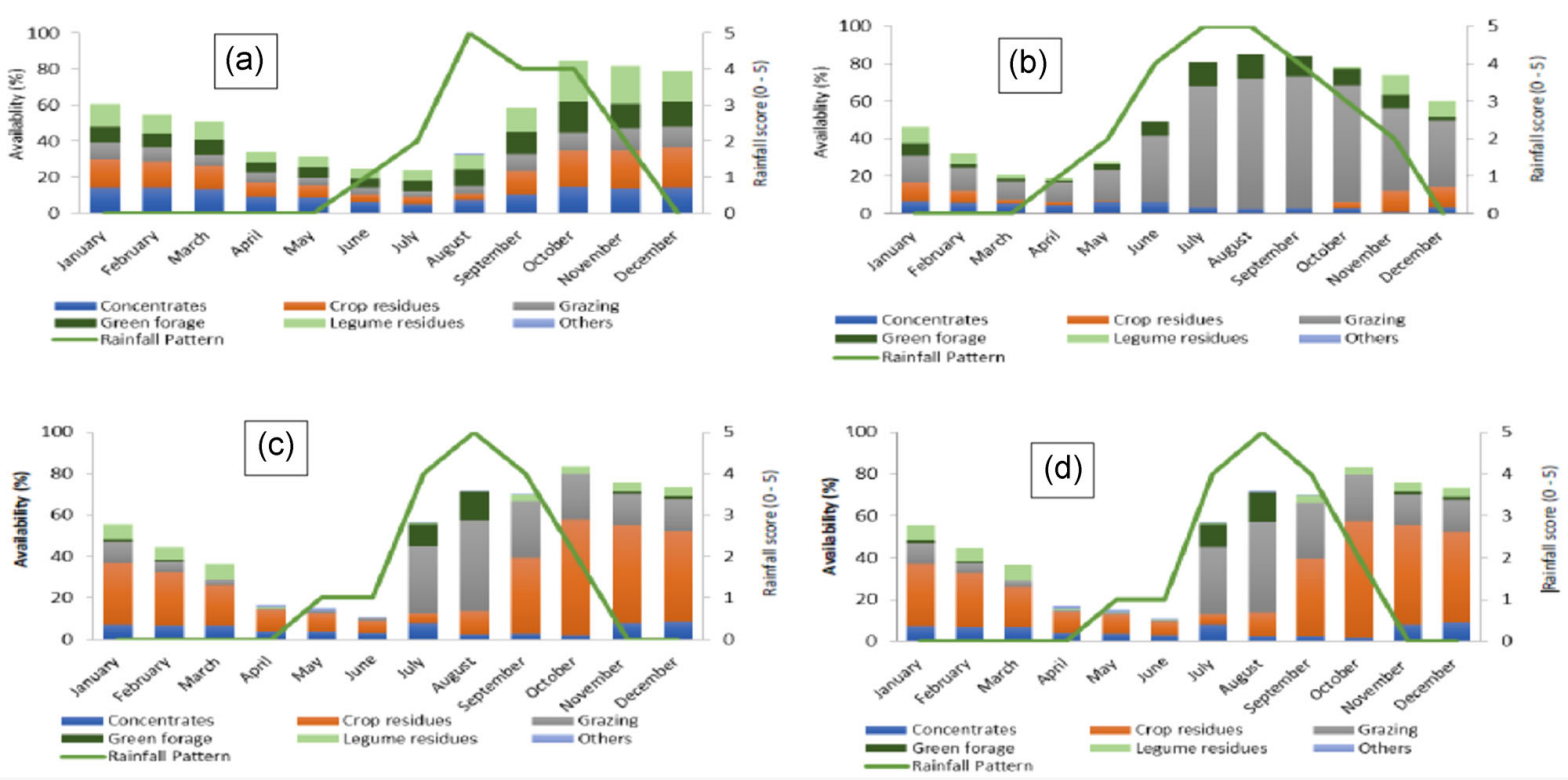
Seasonal variation in the available feed resources in four representative areas of the Sahel: (a) Mahon, Burkina Faso, (b) Thiou, Burkina Faso, (c) Milli, Niger, and (d) Yakubawa, Nigeria (modified from Amole and Ayantunde [2016a] and Amole and Ayantunde, [2016b]). Availability in *y* axis is an average of the scores given by the respondents on availability of different feed resources per month in the community. Rainfall score is an average of the scores given by the respondents on rainfall intensity where 0 = no rainfall and 5 = heavy rainfall

The most common types of feed resources in West Africa include rangelands, crop residues, agro‐industrial by‐products, and browse plants (Bayala et al., 2014). Common forage species for the countries and common challenges are summarized in Table 2. The availability of different feed resources also varies over time. Figure 2 shows the seasonal variation in the availability of different feed types in four representative locations in the West African Sahel.

**TABLE 2:** Common types of feed resources in the Sahel

Type of fodder	Species and varieties	Countries or regions	Common problems or challenges	References
Crop residues	Cereal crop residues–millet [*Pennisetum glaucum* (L.) R.Br], sorghum (*Sorghum bicolor*), maize (*Zea mays* L.), and rice (*Oryza sativa*)	All Sahel	Low quality, bulky and difficult to transport, challenging to process and store, seasonal variability in availability	Amole & Ayantunde (2016b), Zerbo et al. (2016)
	Leguminous crop residues such as cowpea (*Vigna unguiculata* L.) and groundnut (*Arachis hypogaea* L.)	All Sahel	Rapid decline in quality due to leaf losses, bulky and difficult to transport, seasonal variability in availability, high prices	Anele et al. (2011)
Rangeland, herbaceous legumes	False moneywort [*Alysicarpus ovalifolius* (Schumach. & Thonn.) J. Leónard], rat's ear (*Commelina forskaolii Vahl*), dengo (*Zornia glochidiata* Rohb. ex DC.) and *Eragrostis tremula* Hochst. ex Steud., dengo,* Schoenefeldia gracilis* Kunth*, Cenchrus biflorus* Roxb.*, Panicum laetum* Kunth*, *Chinese senna (*Cassia obtusifolia* [now *Senna obtusifolia* (L.) H.S. Irwin & Barneby]), and false moneywort	Southern Sahel	Low quality, high variability in quality and availability depending on season, soil fertility and topography, competition with invasive species	Amole & Ayantunde (2016b), Zerbo et al. (2016)
Rangeland, grasses	*Panicum turgidum* Forssk.*, Aristida sieberiana* Trin. ex Spreng., lemongrass (*Cymbopogon schoenanthus* Spreng.)*, Cyperus jeminicus* Rottb., and *Cyperus conglomeratus* Rottb.	Northern Sahel	Marked variation in quality and intake, limited accessibility for browsing by animals as some may be thorny, restricted intake by animals due to antinutritional factors, seasonal variability in foliage	Bayala et al. (2014), Abdulrazak et al. (2000)
Browse species	Gum acacia (*Acacia senegal*), red acacia (*Acacia seyal* Del.)*, A. laeta*, gum arabic tree (*A. nilotica*), red acacia, gum acacia, twisted acacia (*A. raddiana* Savi), gum arabic tree, kinkeliba (*Combretum micranthum* G. Don), Egyptian balsam [*Balanites aegyptiaca* (L.) Delile], rubber tree [*Calotropis procera* (Aiton) W.T. Aiton], small‐leaved bloodwood (*Pterocarpus lucens* Lepr. ex Guill. & Perr.), dooki (*Combretum glutinosum* Perr. ex DC.), marula [*Sclerocarya birrea* (A. Rich.) Hochst.], Egyptian balsam [*Balanites aegyptiaca* (L.) Delile], hanza [*Boscia senegalensis* (Pers.) Lam. ex Poir.], African myrrh [*Commiphora africana* (A. Rich.) Endl.], African birch [*Anogeissus leiocarpus* (DC.) Guill. & Perr.],and Indian jujube (*Ziziphus mauritiania* Lam.)	Burkina Faso	Generally low quality and intake potential, limited accessibility as some may be thorny, restricted intake by animals due to antinutritional factors, seasonal variability in foliage	Zampaligré et al. (2013)
Introduced forages and pasture (includes cultivated pasture)	Caribbean stylo [*Stylosanthes hamata* (L.) Taub.]*, Digitaria umfolozi* D.W. Hall, Indian goosegrass [*Eleusine indica* (L.) Gaertn.], finger millet (*Eleusine coracana* Gaertn.), buffel‐grass (*Cenchrus ciliaris* L.)*, Mucuna atropurpurea* (Roxb.) DC. ex Wigh, *Mucuna aterrina, Scaphispatha gracilis* Brongn. ex Schott, cowpea [*Vigna unguiculata* (L.) Walp.]*, Lablab purpereus* (L.) Sweet, Congo grass (*Brachiaria ruziziensis* Germ. & C.M. Evrard), guinea grass (*Panicum maximum* Jacq.), and hyacinth bean (*Dolichos lablab* L.)	Mali, Burkina Faso	Seed availability, high price of seed, limited technical know‐how by farmers on production and conservation, availability of land to cultivate the species, low adoption by farmers	Bayala et al. (2014), Sanon & Kanwé (2002), Kouame et al. (1992)
Fodder banks	Jumbay [*Lucaena leucocephala* (Lam.) de Wit], white mulberry (*Morus alba* L.), quickstick [*Gliricidia sepium* (Jacq.) Steud.]*, Acacia* spp., jumbay, mango (*Mangifera indica* L.)*, Musa* spp., pigeon pea [*Cajanus cajan* (L.) Millsp.]*, Tamarindus indica* L., stylo [*Stylosanthes guianensis* (Aubl.) Sw.], butterfly pea (*Centrosema pubescens* Benth.)*, Desmodium* spp. etc . In Burkina Faso, kosso (*Pterocarpus erinaceus* Poir.), and *Pterocarpus lucens* Lepr. ex Guill. & Perr.	Mali, Burkina Faso and Niger	Very high cost for initial establishment, limited biomass productivity, competition with crop land, limited knowledge on requirements for establishment, harvest, management etc., very little participatory on‐farm research has been conducted on fodder banks	Bayala et al. (2014), Sib et al. (2016, 2020), Vall et al. (2019)
Agro‐industrial by products	Cotton seed cake, bran of millet, sorghum, maize, wheat, and concentrate feeds		Toxicity, for example, due to aflatoxin, spoilage when poorly conserved, absence of quality control, limited availability in rural areas	FAO (2014)

### 3.2 Rangelands

Rangelands are the main source of feed for animals in the Sahel. The rangelands of the Sahel are composed of herbaceous plants dominated by annual species (more than 80%), and a scattered population of shrubs and trees (Hiernaux & Le Houérou, 2006). The species composition of rangelands differs along the agroecological gradient within the Sahelian region, with rangelands in the northern part of the Sahel being composed of thorny shrubs and trees (Bayala et al., 2014). In the southern Sahel, rangelands are dominated by herbaceous grasses with a denser tree and shrub layer (Hiernaux & Le Houérou, 2006).

The quality, availability, and biomass production of rangelands in the Sahel vary by geography, topography, season, and climate. This is associated with both the temporal and spatial distribution of precipitation (Bayala et al., 2014), with abundance and better nutritive value of forages during the rainy season, and scarcity and poor quality during the dry season (Konlan et al., 2018). Available biomass in the Sahelian zones of Mali and Senegal allows a voluntary feed dry matter (DM) intake of 1.8 to 2.7 kg DM 100 kg^–1^ of live weight (LW) for cattle, 1.7–3.2 kg DM 100 kg^–1^ of LW for sheep, and up to 6 kg DM 100 kg^–1^ of LW for goats (Dicko et al., 2006; Diop et al., 2005). However, wide local variation in herbage production due to differences in soil type and rainfall has also been reported within the Sahel region (Wylie et al., 1995). Annual mean herbage production ranges from 600 kg DM ha^−1^ in the northern Sahel with 200 mm of rainfall to 2,400 kg DM ha^−1^ in the southern Sahel with 600 mm of rainfall (Glatzle, 1992). In a report from the Diffa administrative district (northern Sahel) in Niger, annual herbage production varied from 305 to 936 kg DM ha^−1^ among various sites within the same district (Wylie et al., 1995). Similarly, Penning de Vries and Djiteye (1982) reported annual herbage production of rangeland from 1.1 to 4 Mg DM ha^−1^ across the rainfall gradient in West Africa. Within the same site, variations also exist due to runoff water patterns, topography, geomorphology, and plant species (Breman & de Ridder, 1991). The nutrient composition of herbage produced, is often inversely proportional to available soil water during the growing season (Breman & de Wit, 1983). In northern Niger at 320 mm rainfall, steers gained up to 80 kg of LW per animal during the short rainy season and lost little weight during the dry season provided sufficient forage to meet maintenance requirements was available (Klein, 1981; Wylie et al., 1983). In contrast, cattle grazing rangelands receiving 400–500 mm further South, gained 60–90 kg LW during the rainy season, but net annual gains per head were much lower due to weight losses of 30–50 kg LW during the dry season (De Leeuw & Tothill, 1990).

### 3.3 Improved and cultivated pastures

Many exotic species have been introduced in the Sahel and screened on‐station based on their yield potential, nutritional composition, fertilizer requirements, persistence, management, and use as forage legumes (Bayala et al., 2014; Birie‐Habas, 1965; Kouame et al., 1992; Sanon & Kanwé, 2002; Thomas & Sumberg, 1995). Most improved forage species were introduced only at experimental station level and there is a lack of information on integration of these practices into the farmers’ livestock systems in the Sahel (Bayala et al., 2014), the resulting livestock productivity gains and the willingness of farmers to pay for these interventions. This has also contributed to limited adoption of improved cultivated forage species across the Sahel (Thomas & Sumberg, 1995).

Other factors also contribute to the limited adoption of improved or cultivated forage species in the Sahel. These include scarcity of cropland, which makes cereal or food crop cultivation to be a priority over forage crop cultivation, as well as overlapping cropping seasons with food crops thus creating competition for land, labor, and other inputs. Moreover, there is very limited supply of quality forage seeds, which further limit adoption of forage cultivation among most smallholder livestock producers in the Sahel (Onana et al., 2000).

### 3.4 Fodder banks and their adoption in the Sahel

Fodder bank is a system of forage cultivation where forage species mostly woody and sometimes herbaceous leguminous species (sometimes mixed) are strategically planted on degraded farmlands (ESGPIP, 2017). Usually established to rehabilitate farmlands while providing source of protein for livestock, fodder banks can be grazed or used in cut‐and‐carry systems (ESGPIP, 2017). Introduced and indigenous leguminous fodder trees and shrubs are commonly used fodder species for establishment of fodder banks (Table 2). In Burkina Faso, high density (20,000 plants ha ^−1^) fodder banks of jumbay [*Leucaena leucocephala* (Lam.) de Wit] and white mulberry (*Morus alba* L.) were able to quickly provide fodder within a period of 12 mo with initial outputs of 4–10 Mg DM ha ^−1^) (Sib et al., 2016; Sib et al., 2020). A mixture of kosso (*Pterocarpus erinaceus* Poir.) and quickstick [*Gliricidia sepium* (Jacq.) Steud.] established as fodder banks and pruned at 50 cm above soil surface have produced 8 Mg fresh weight ha^−1^ (Niang et al., 2002).

Despite the potential of fodder banks, the practice is not widely practiced by farmers and the level of adoption by farmers is low. The main reasons for limited adoption include lack of knowledge on establishment, management, and utilization, which is mainly caused by limited or no participatory research (Bayala et al., 2014). Specifically, there is scarcity of technical knowledge on indigenous fodder trees and shrubs (Balehegn, 2017; Bayala et al., 2014). The other reason for low adoption is land tenure systems in the Sahel, which do not allow establishment of fodder banks on a leased land as only annual crops can be cultivated on such land. Besides there is no comparative advantage in establishing fodder banks when compared to growing annual crops, which is critical to food security of the household. In the 1980s the cost of establishing fodder banks stood between US$200 and $500 ha^−1^, making it unaffordable for most smallholder farmers (De Montgolfier‐Douèvi, 1980). In some cases, fodder banks have not been economically feasible, compared to the traditional practice of lopping trees from the wild, showing negative net present values, especially with low tree densities (Hamer et al., 2007).

### 3.5 Rangeland browse plants

Browse plants refer to woody perennials, trees, shrubs, and dwarf shrubs that browsing ruminants, mainly goat and sheep, feed on. They form an important component of the livestock diet, particularly in the dry season (Le Houérou, 1988). The most common browse and fodder trees in Sahel include *Acacia* and *Commiphora* spp. (Table 2). Most browse species occur naturally or are introduced and managed in silvopasture on range and pasturelands and their management requires little or no labor, time, technical know‐how or resources relative to crop farming. Unmanaged silvopastures are common practice across the Sahel, as trees are an integral part of grazing lands. Silvopastures improve soil fertility, forage quality, productivity and as a result animal productivity, as well as CO_2_ sink of grazinglands as compared to conventional, grass‐dominated pasture lands (Bayala et al., 2007; Coulibaly et al., 2020). For instance, phosphorus and nitrogen availability has been observed to be higher under canopy trees than in areas beyond canopies in a white acacia [*Feidherbia albida* (Delile) A. Chev.]–shea tree (*Vitellaria paradoxa* C.F. Gaertn.) woody rangeland in Burkina Faso (Gnankambary et al., 2008). The multipurpose nature of browses as fuelwood, shade, food (fruits), poles, etc. as well as their potential to improve soil fertility and conservation are added advantages. In terms of utilization as animal feed, browse trees currently play an important role, as animals under confinement often receive one type or another of browse, from fallow lands or around homesteads (Zampaligré et al., 2013). Browses are also specifically important during the dry season when feed reserves are depleted (Zampaligré et al., 2013). Efficient complementation of browses with pastures and crop residues needs to be researched, to exploit their potential nutritive value. Browsers, particularly goats, can consume various parts of woody plants such as leaves, twigs, thorns, bark, wood, bulbs, tubers, roots, flowers, seedpods, and fruits (Lamidi, 2004; Le Houérou, 1988). Browse could, therefore, supplement the low energy and protein content of herbaceous pasture vegetation and crop residues during the dry season. Trees are more reliable than herbaceous legumes and grasses for providing high‐quality protein supplement in the dry season (Arigbede et al., 2007). Although the total number of browse species identified in the Sahel (more than 60) is high (Bayala et al., 2014), only a few species contribute significantly to daily browsing time of ruminants. Commonly utilized browse species are listed in Table 2.

### 3.6 Crop residues

Crop residues found in West African Sahel include stover of cereals such as millet [*Pennisetum glaucum* (L.) R.Br], sorghum [*Sorghum bicolor* (L.) Moench], maize (*Zea mays* L.), haulms of leguminous crops such as cowpea [*Vigna unguiculata* (L.) Walp.] and groundnut (*Arachis hypogaea* L.), and rice (*Oryza sativa* L.) straw. Large quantities of residues are produced every year in the different Sahelian countries in the Sahel. In 2010, the total quantity of cereal straws was estimated at about 80 million Mg for all the countries of the West African Economic and Monetary Union (known by its French acronym, UEMOA), which include Benin, Burkina Faso, Ivory Coast, Guinea‐Bissau, Mali, Niger, Senegal, and Togo.

An important limitation of crop residues is their low feeding value, mainly low metabolizable energy, and low protein and mineral contents, which cannot support adequate rumen microbial growth or meet the nutritional requirements for increased livestock performance (Simbaya, 2002). Crop residues are high in fiber and lignin, consequently, they are poorly degraded in the rumen. Their bulk contributes to gut fill, and this coupled with their low nitrogen and mineral concentrations, results in very low intakes.

Similar to other feed types, the availability of crop residues varies seasonally (Fernandez‐Rivera et al., 2005). Usually crop residues are abundant at the end of the growing season and the stocks dwindle with duration of the dry season (Fernandez‐Rivera et al., 2005) (Figure 2). The lack of storage facilities also limits the use of crop residues in smallholder livestock production systems (Simbaya, 2002). Crop residues are bulky by nature; therefore, storage, handling, transporting, and processing requires significant investment and labor.

The distribution of crop residue production does not necessarily correspond with animal distribution, causing a shortfall in some areas and a surplus in others (Simbaya, 2002). According to Williams et al. (1997), availability of crop residues at the farm level depends not just on production levels but also on a variety of social and economic factors, cultural practices, the use of modern crop varieties and the opportunities for market and nonmarket exchanges. Interannual fluctuations in rainfall can also affect crop residue yield, which may in turn affect the ratio between edible and nonedible fractions within residues.

### 3.7 Cereal crop residues

Millet and sorghum are the most dominant crops in the West African Sahel due to their productivity and drought tolerance. The stems, sheaths, and leaves are usually referred to as stovers and are used as livestock feed in the region. Traditionally, pastoralists exchanged manure and milk for grains (Ayantunde et al., 2011) or for access to graze crop residues (millet, sorghum, maize, and rice) on farmers’ fields after grain harvest. By doing so, the farmers benefited from the manure deposited by the animals during grazing of crop residues. Cereal residues may either be harvested or left in situ on the field to be grazed. In a study in the Sudanian zone of Burkina Faso, Andrieu et al. (2015) confirmed that farmers left around 80% of cereal crop residues on their fields. However, other uses exist. Cereal crop residues are sometimes left on the field to be used as mulch, transported to the homestead for stall feeding, used as fencing, building, or roofing materials, or as fuel (Andrieu et al., 2015).

In terms of quality, depending on location, stage at harvest and other factors, a crude protein (CP) concentration of 78 g kg^−1^ with a digestibility coefficient of 504 g kg^−1^ was reported for millet stover (Powell & Fussell, 1993). However, the value has ranged from 30 to 50 g kg^−1^ CP in another study (Nantoumé et al., 2000) and a range of 27–57 g kg^−1^ was reported for sorghum stover in the Sahel (Kiema et al., 2008). Millet ranks first in terms of residue biomass, accounting about half of the cereal residues, followed by sorghum (Abdoulaye & Ly, 2014).

The spatial distribution of these residues follows the agroecological gradients and the particular crops primarily grown in those zones. In most dry areas, millet is grown more often than sorghum and maize (Amole & Ayantunde, 2016a), while maize is more abundant in the moister zones within the West African Sahel. According to Savadogo et al. (1999), the North Sudano–Sahel region has the highest potential to produce feed resources in the form of crop residues. Livestock either owned or contracted are allowed to graze these residues in the field.

Most of the crop residues are abundant during the months of October–December (at the end of the rainy or cropping season) and are in greatest use during the dry season when the available pasture is low in quantity and quality (Figure 2). The quantity of cereal residue available at a given time will depend on the area of cropped land, the crop cultivars chosen, and management practices that influence the yield. Besides, the quantity of crop residues used by the farmers will depend on some interacting factors such as farmers' preferences, household labor, crop production levels, access to alternative biomass resources; livestock management practices, and biomass demand (de Leeuw, 1997).

### 3.8 Legume haulms

Common legume crop residues in the Sahel include cowpea hay and groundnut haulms (Ayantunde et al., 2007; Fall et al., 2005). Cowpea hay is an important fodder source for livestock in crop–livestock systems in the Sahel region of West Africa (Agyemang, 2012). The legume haulms are normally harvested and conserved either as dry‐season feed for the farmers' animals or for sale to other farmers during the critical period of feed scarcity in the mid‐ to late dry season (Singh & Tarawali, 1997). Cowpea hay can provide adequate protein and energy to sustain ruminant production during an extended dry season (Anele et al., 2011). From various experiments in Niger, feeding levels between 300 and 600 g DM day^−1^ of cowpea hay and 400 g DM day^−1^ of millet bran were established for profitable sheep fattening, along with ad libitum feeding of roughage such as bush hay or millet stover (Fernández‐Rivera et al., 2005). Feeding trials by Savadogo et al. (2000) showed that the intake of cowpea hay by sheep was 86 g organic matter (OM) kg^−1^ LW^0.75^ d^−1^, and the selective consumption of leaves results in higher intakes of protein and digestible OM than expected from the offered haulms. In sheep fed 200–400 g DM d^−1^ of cowpea haulms as a supplement to a basal diet of sorghum stover in northern Nigeria, the resulting average LW gain (80 g DM d^−1^) was twice that obtained with sorghum fodder alone (Singh et al., 2003).

Variations in the nutritional profile of crop residues across West Africa are to be expected as nutrient compositions of forages and agro‐industrial by‐products vary over time due to different factors such as stage of harvest, stems/leaf ratio, period and mode of storage, cultural practices, harvesting practices, and soil fertility (Adebowale, 1988).

### 3.9 Agro‐industrial by‐products

Agro‐industrial by‐products (AIBPs) are waste or co‐products arising from the processing of crop or animal products by industries while some other agro‐by‐products are mainly from household processing of some agricultural products (Sindhu et al., 2002). The use of AIBP as livestock feed can reduce the cost of production, improve the quality of feed, ensure regular feed supply even during the period of scarcity, and ultimately increase the profit margin of livestock farmers (Sindhu et al., 2002).

Generally, AIBP are either energy, protein, or combined protein–energy sources (Aregheore, 2000). Such energy sources are rich in fermentable carbohydrates and low in protein. A few examples are molasses, a by‐product of the sugar industry (41 g CP kg^−1^ DM and 12.7 MJ GE kg^−1^ DM), while cereal by‐products include brewers' spent grains and bran from wheat (*Triticum aestivum* L.), rice, and maize (Cheeke, 1991). Some are protein‐rich products such as cakes and meals. Examples are soybean [*Glycine max* (L.) Merr.] meal, which can contain 480 g kg^−1^ crude protein (CP) (Obese, 1998), palm kernel meal (180 g CP kg^−1^ DM CP), copra meal (188 g kg^−1^ DM CP), cocoa husk (Otchere et al., 1986), and cotton (*Gossypium hirsutum* L.) seed, which is perhaps the regionally most dominant by‐product with an average CP content of 249 g kg^−1^ DM. Local cereal brans (millet bran, sorghum bran) are examples of household agro‐by‐products. Sahelian countries were depicted as the largest producers of cereal bran in West Africa (FAO, 2014).

The availability of agro‐industrial by‐products varies with time and place due to specificity of crops grown in different areas and times. This also results in variation in prices. Beside the constraints of seasonality and price, there are several anti‐nutritional factors, including various toxic compounds, which are deleterious to animal health and performance for some of agro‐industrial by‐products (Aregheore, 2000). Gossypol, a polyphenolic pigment present in the kernel and coat of the seed has been reported in many cotton by‐products, while mycotoxin contamination, mostly commonly with aflatoxin produced by *Aspergillus flavus*, been reported for groundnut cake (FAO, 2014).

Despite the availability of these feed resources in the Sahel, few if any of the Sahelian countries have comprehensive feed databases that catalogue the types of feed available, their yields, nutritional qualities, seasonality of production, prices, and accessibility. Such feed databases are critical for national inventories to plan for external shocks like drought or conflict that affect significant imports from neighboring countries and alter local availability of feeds. They are also critical for developing and using ration formulation software and apps to ensure that least cost, climate‐smart diets are fed to optimize livestock productivity. Such a database for Burkina Faso has been created by the Feed the Future Innovation Lab for Livestock Systems and it already lists more than 3,800 feeds. This data base will be posted with unrestricted access on the website of the government agriculture research agency: Institut de l'Environnement et de Recherches Agricoles (INERA; Environmental Institute for Agricultural Research).

### 3.10 Nutritive value of the different feed resources in the Sahel

The quality of feed resources, especially rangelands and crop residues in West African Sahel, varies seasonally (Bayala et al., 2014). Generally, the quality of rangelands is best in the wet season and then declines rapidly as the season advances from wet to dry (Ayantunde et al., 2000). The annual herbaceous pasture species are generally of higher quality in terms of CP and organic matter digestibility than the perennial herbaceous species (Ayantunde et al., 2000). The nutritive value of crop residues generally depends on crop varieties, harvest period, cultural practices, soil fertility, conservation method, and feeding practices (Smith, 1988). The leaves and stems of the various legumes provide excellent protein supplementation to livestock in the Sahel. Cowpea hay, for example, has been reported to contain CP values of 77–217 g kg^−1^ (Kaasschieter et al., 1998; Savadogo et al., 2000) and is often used as a supplement for the low‐quality animal feed crop residues or rangeland. The dry matter digestibility of cowpea hay is about 650–700 g kg^−1^ with leaves having a digestibility between 600 and 750 g kg^−1^, while stems have a digestibility between 500 and 600 g kg^−1^ (Karachi & Lefofe, 2004; Savadogo et al., 2000). Table 3 presents the proximate nutrient composition of various crop residues in the West African Sahel. These results represent only a few of several results documented in West Africa as some authors such as Bayala et al. (2014) have presented a review of the fodder quality in the Sahel.

**TABLE 3:** Nutrient composition, metabolizable energy (ME), and in vitro organic matter digestibility (IVOMD) of available crop residues in Burkina Faso

	Nutrient concentration						
Crop residues	**Ash**	**CP**	**NDF**	**ADF**	**ADL**	ME	IVOMD
	g DM kg^–1^					MJ kg^–1^	g DM kg^–1^
Cowpea haulms^a^	92	170	459	242	60	9.22	648
Groundnut haulms^a^	137	117	452	447	152	7.26	539
Millet stover^a^	75	149	641	711	147	5.24	350
Maize stover^a^	53	44	672	425	77	7.88	556
Maize husk^a^	59	19	871	431	50	8.86	579
Sorghum stover^a^	44	14	861	648	120	5.57	375
Cowpea haulms^b^	120	100	456	726	393	7.68	555
Groundnut haulms^b^	109	207	129	355	302	4.97	651
Millet stover^b^	134	36	597	399	629	6.07	441
Sorghum stover^b^	107	40	736	482	817	6.9	467

*Note*. ADF, acid detergent fiber; ADL, acid detergent lignin; CP, crude protein; DM, dry matter; NDF, neutral detergent fiber.

^a^ Amole and Ayantunde (2016a).

^b^ Amole and Ayantunde (2016b).

Browses are generally higher in CP (Table 4) than crop residues and could therefore, supplement the low energy and protein content of grass forage if used effectively during the dry season. Trees are more reliable than herbaceous legumes and grass in providing high‐quality protein supplement in the dry season (Bayala et al., 2012). The high tannin content in browses is a major limitation to their utilization as ruminant feeds. Moreover, their availability is seasonally variable only a limited amount of biomass that can be harvested by browsers.

**TABLE 4:** Nutrient composition, metabolizable energy (ME) and in vitro organic matter digestibility (IVOMD) of some browse plants in Burkina Faso

	Nutrient concentration						
Browse	**Ash**	**CP**	**NDF**	**ADF**	**ADL**	ME	IVOMD
	g DM kg^–1^					MJ kg^−1^	g DM kg^−1^
African mahogany [*Khaya senegalensis* (Desr.) A.Juss.] (leaf)	127	149	425	347	74.2	7.17	522
Drumstick tree (*Cassia sieberiana* DC.) (pods)	48	81	325	316	107	9.09	610
Camel's foot tree [*Piliostigma tonenji* (Schum.) Milne‐Redh.] (pods)	116	77	555	535	239	6.65	469
Assyrian plum (*Cordia myxa* L.) (leaf)	141	151	595	672	279	5.54	423
African copaiba balsam tree [*Daniellia oliveri* (*Rolfe*) Hutch. & Dalziel] (leaf)	64	189	569	422	137	10.2	705
Mango (*Mangifera indica* L.) (leaf)	86	82	450	389	116	6.52	458
Drumstick tree (*Cassia sieberiana* DC.) (leaf)	43	133	665	554	249	7.15	501

*Note*. ADF, acid detergent fiber; ADL, acid detergent lignin; CP, crude protein; DM, dry matter; NDF, neutral detergent fiber. Source: Amole and Ayantunde (2016a).

### 3.11 Effect of climate change on feed resources in West African Sahel

Climate change and variability is a major challenge to livestock production in West African Sahel and this is expected to affect livestock at both species and breed levels (Amole & Ayantunde, 2016c; Zougmore et al., 2016). Drought is particularly a major constraint to livestock production in the region where there has been changing frequency of extreme climate conditions (Zougmore et al., 2016). Specific effects of climate change on livestock in West Africa region as reported by Zougmore et al. (2016) may include changes in availability and quality of feed resources, access to water, species and breeds of livestock that can be kept, livestock mobility, and animal diseases.

Climate change effects on forage availability and quality in West African Sahel include changes in herbage growth, changes in floristic composition of vegetation, changes in herbage quality, and changes in importance of crop residues as animal feed (Thornton et al., 2009). These likely effects of climate change on feed resources will be modified by soil fertility, changes in land use, and grazing management (Hiernaux et al., 2009). Generally, the effects of climate change on herbage growth will depend on the plant species as increase in future CO_2_ levels may favor C3 plants over C4 plants while the opposite is expected under associated temperature increases (Thornton et al., 2009; Zougmore et al., 2016). The likely effects of rainfall on herbage yield may be associated more with extreme rainfall events (droughts and floods) than the mean annual precipitation. For example, long‐term vegetation monitoring (1994–2006) in southwestern Niger showed that there is a weak correlation between annual precipitation and herbage yield (Hiernaux et al., 2009).

The same trend was also found for correlation between annual precipitation and floristic composition of the monitored vegetation sites. The likely effects of increased temperature on forage quality will be lignification of plant tissues and the associated decline in digestibility and the rates of degradation in the rumen. This will lead to reduced nutrient availability for animals and ultimately will lead to reduction in livestock production. Increased frequency and severity of extreme climate events such as droughts and the associated acute feed scarcity will decimate livestock as recorded in the droughts of early 1970s and 1980s in the Sahelian countries (Turner, 2000). Apart from high herd mortality, droughts will change the livelihood options of poor livestock keepers as was the case in the past four decades when droughts compelled many pastoralists in the Sahel to sedentarize and grow crops, and some to move out of livestock rearing completely (Zougmore et al., 2016). Increasing sedentarization by the pastoralists to grow crops in addition to livestock rearing will increase feed biomass from crop residues and this trend of growing importance of crop residues as animal feed in West African Sahel has been reported by Ayantunde et al. (2018). Options to better adapt feed resources to climate change effect may include cultivation of resilient forage species, fodder conservation including silage and hay making, improvement of forage quality such as processing of locally available feed resources, particularly crop residues, integration of forage legumes into arable crops and grazing management (Amole & Ayantunde, 2016c). These feed interventions can improve livestock productivity, enhance adaptation, and reduce greenhouse gas emissions.

### 3.12 Feed marketing

**FIGURE 3:**
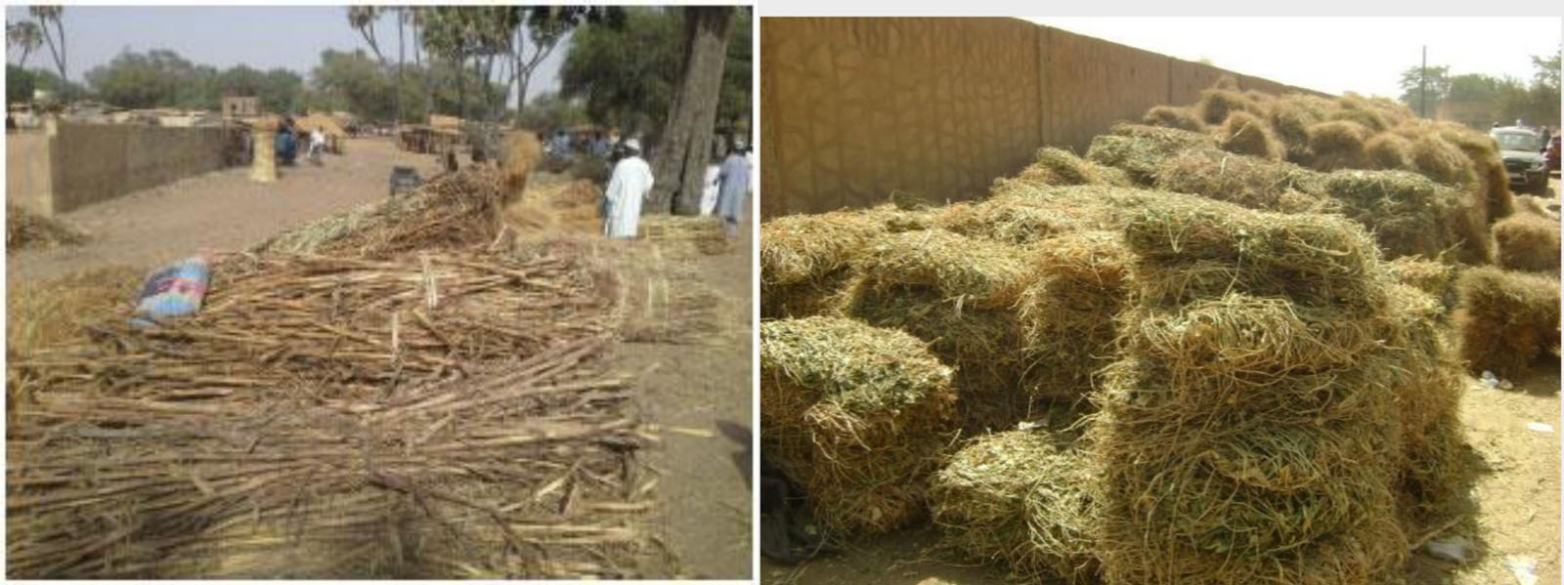
Open air crop residue marketing in West African countries

Feed marketing in the West African Sahel exists at many levels. Crop residues are sold either directly off the farms to nearby livestock farmers or transported and sold as bundled residues along roadsides and in or around livestock markets and peri‐urban communities (Figure 3) (Grings et al., 2010). These feed markets are normally situated near livestock markets, and they mainly sell crop residues such as cowpea hay and groundnut haulms, agricultural by‐products such as cottonseed cake and cereal bran, concentrate feed from small‐scale feed industry, and leaves from various shrubs and green fodder harvested from the rangelands. Ayantunde et al. (2014) reported that feed markets have sprung up in many cities of West African Sahel in response to growing livestock populations in peri‐urban areas. In a report from Yatenga province of Burkina Faso, Traore (2016) confirmed the existence of fodder markets with many actors (collectors, wholesalers, retailers, transporters) supporting their livelihood with fodder marketing.

The sale of crop residues (cowpea hay, groundnut haulms, cakes, and bran) is a major source of household income to crop farmers (Ayantunde et al., 2007; Williams et al., 1997). In terms of types of fodder marketed, Abdoulaye and Ly (2014) reported that cereal straw is virtually never traded, especially in semi‐arid zones, where it is left on the field unless used by the household (for fuel, construction, etc.). However, this trend as reported by Abdoulaye and Ly (2014), may be limited to rural communities where most households grow cereals and often leave the residues on the field for grazing by animals. Others have reported sales of cereal residue in Burkina Faso and Niger (Amole & Ayantunde, 2016a, 2016b). We noted that price of crop residue varies in spatial and temporal dimensions, and in particular, that of legumes varies according to seasonal availability (Grings et al., 2010). The highest price was reported during the wet season (June–September) and the lowest immediately after harvest (October and November). Other agricultural by‐products, such as cottonseed cake and cereal bran, and of concentrate feeds did not vary significantly across the seasons, suggesting adequate year‐round availability (Ayantunde et al., 2014). Poor storage methods or lack of facilities limits year‐round availability of crop residues especially cowpea haulms. Grings et al. (2010) reported that improved storage methods will decrease leaf loss after harvest and could improve the market value of cowpea haulms over a longer period of the year.

The survey on the emerging feed markets for ruminant production in urban and peri‐urban areas of northern Ghana by Konlan et al. (2018) revealed that there were seasonal variations in the quality of feed resources in the sampled areas. For instance, CP concentration of groundnut haulm was 98 and 122 g kg^−1^ in the early dry season and late dry season, respectively. Notable variations were also recorded for fiber, metabolizable energy, and digestibility of the identified feed resources in Ghana.

**FIGURE 4:**
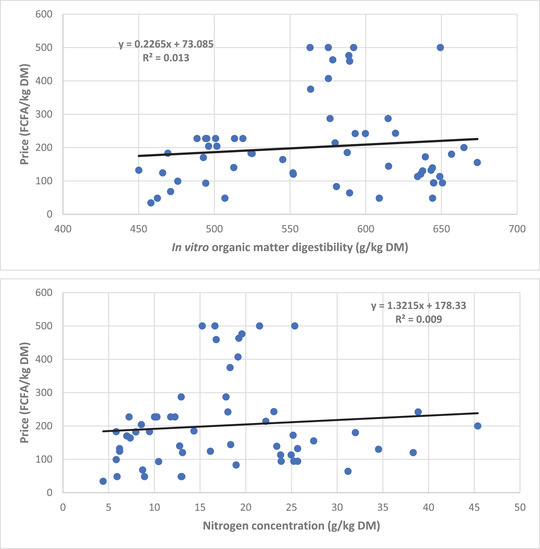
Relationship between, respectively (top) price and in vitro organic matter digestibility and (bottom) price and nitrogen concentration (bottom) of livestock feeds sold in markets at Ouagadougou, Burkina Faso, in the late dry season (Ayantunde, 2020). FCFA, Franc Communauté Financière Africaine (US$1 = 550 FCFA during the market survey period); DM, dry matter

Another important problem with feed marketing in West Africa is the lack of relationship between price and quality. Studies in different parts of Burkina Faso (Ayantunde, 2020), Niger (Jarial et al., 2017), and Mali (Ayantunde et al., 2014) have shown little to no relationship between the cost and nutritional value of livestock feeds, though a few exceptions exist. For example, from the feed market surveys conducted in Burkina Faso between April and May 2019 (late dry season), there was no correlation between price and quality (nitrogen concentration and in vitro organic matter digestibility) in the Ouagadougou peri‐urban area (Figure 4) (Ayantunde, 2020). However, there was a significant positive relationship between price and nitrogen concentration in the market at Dori, a rural town. Feed quality standards are either nonexistent or hardly used and most forages are sold based on their bulk, organoleptic characteristics, or perceptions of the quality by the buyers.

### 3.13 Knowledge gaps on feed resources in the West African Sahel

#### 3.13.1 Understanding of supply demand trends

**FIGURE 5:**
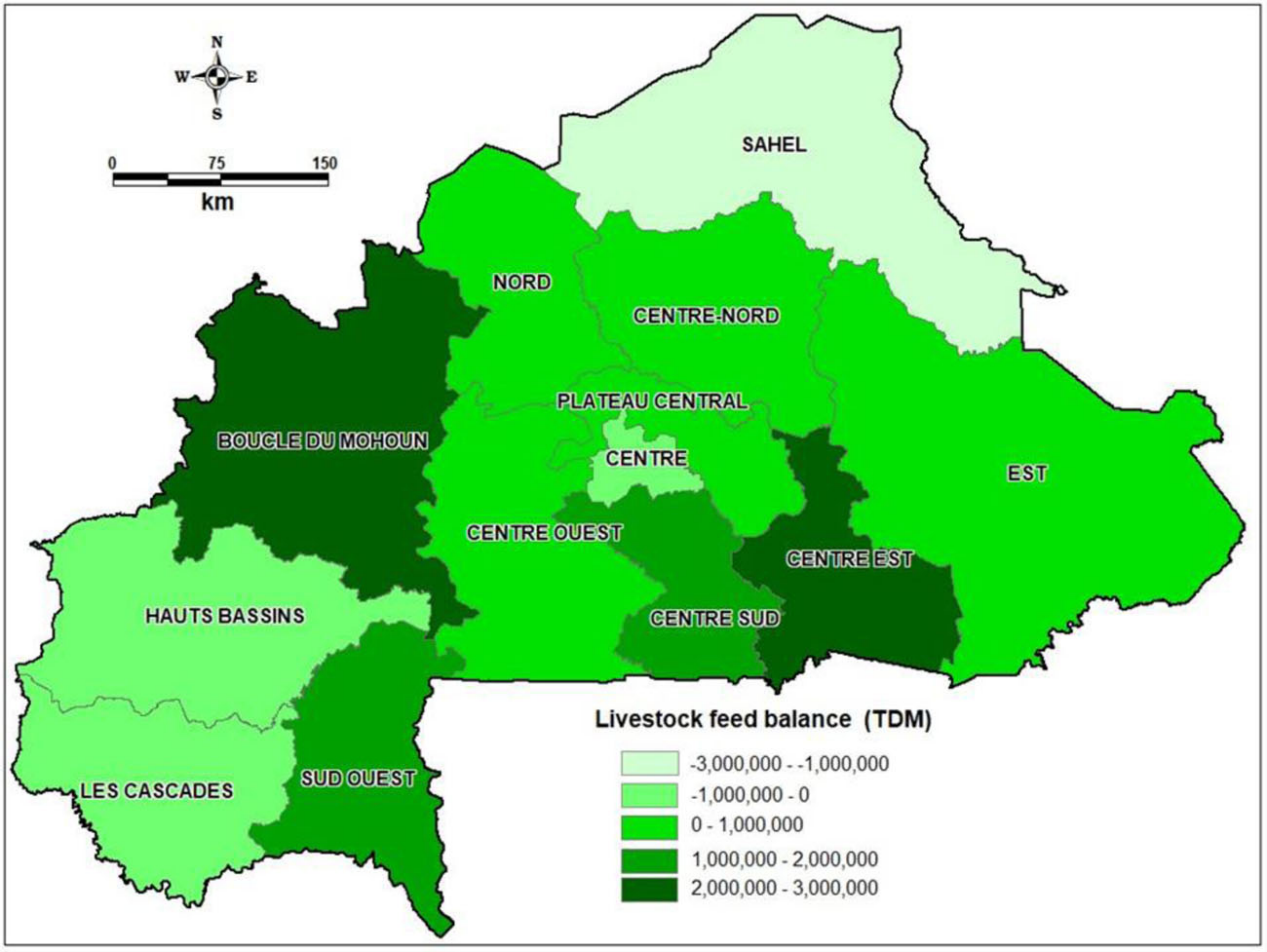
Livestock feed balance in tonne dry matter (TDM) at regional level in Burkina Faso in 2019

In most of the reviewed publications, feed scarcity is presented as a general problem without adequate information about the trends in supply and demand. For instance, a recent analysis of feed demand and supply in Burkina Faso revealed that at the national level there was a feed surplus of up to 6 million Mg of DM, yet there was a feed deficit of 2 million Mg of DM in the Sahel region, and lower deficits in three other regions (Figure 5) (LSIL, 2021). Without such information, appropriate long‐term strategies for addressing problems of feed supply and feeding challenges cannot be developed and implemented. Information on trends in feed supply and demand is also necessary to avoid misleading assumption on inadequate feed supply based on the evidence of feed purchase at specific location.

#### 3.13.2 Limited information on efficacy of feed improvement technologies

The efficacy and importance of technologies for feed quality improvement are not well understood with respect to conservation, storage, and processing of different feed resources to mitigate spatial and temporal feed supply, and demand problems. In most of the study region, livestock production and the target interventions are not market‐oriented, consequently productivity indices are not well defined to evaluate effects of interventions. In addition, limitations in knowledge and skills on various aspects of feed production, processing, quality assessment, and marketing has been identified in Niger and Burkina Faso (Amole & Ayantunde, 2016a, 2016b).

**FIGURE 6:**
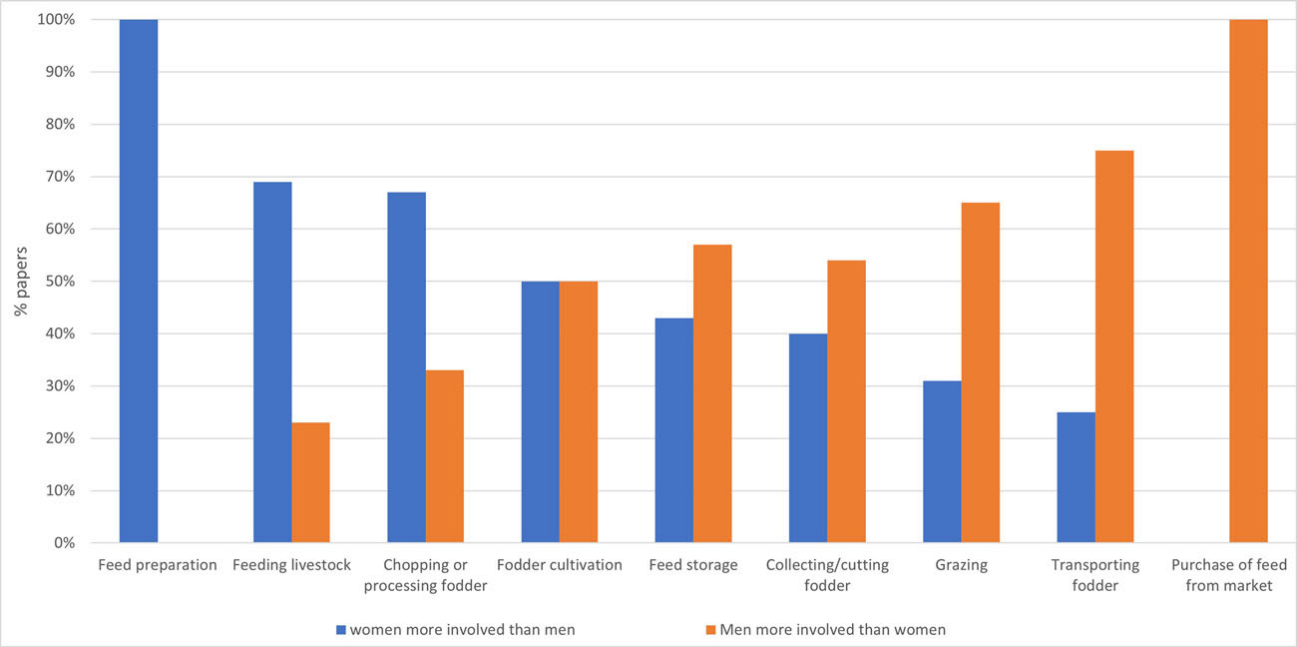
Gender labor division in livestock feeding (results from review of 44 studies by Harris‐Coble et al. [2021])

There is a limited understanding and underappreciation of gender issues in feed resources supply: Despite their considerable involvement and contribution, for instance in the feed sector (Figure 6), women's roles in livestock production have been underestimated and widely underreported. Women face several challenges in accessing and having control over the resources that are linked to fodder and feed production (Baltenweck et al., 2020). These barriers stem from several cultural and socio‐economic issues. Across the Sudano–Sahelian region, women's livestock ownership and decision making about the livestock production operation at the household level is constrained by cultural gender‐biased relationships (Turner, 1999). Women are often constrained by the availability of manual labor because feed handling and improvement techniques involve labor, which can be a constraint to women. Access to training can also be problematic, either because of their existing workloads or because they require consent from their husbands to participate. Interventions supposed to solve gendered problems in the livestock sector do not always achieve the intended purpose, in fact, in some cases may exacerbate problems of gender equity. For instance, in Burkina Faso, seven sustainable intensification interventions have been judged to have low or no effect on gender equity by more than 50% of 400 respondents in two counties (Ayantunde et al., 2020). A forage chopper introduced in Tanzania resulted in unintended increase in labor required by women, despite the intention otherwise (Fischer et al., 2017, 2018). Gendered challenges in the adoption of livestock feed technologies reflect gendered difference in resource access and ownership. A study in Ghana identified that gendered differences in access to resources such as land, water, fertilizers was a reason for gender‐based difference in the adoption of improved maize varieties (Doss & Morris, 2000) Ensuring women's access to extension services, knowledge, credit, and technologies is, therefore, critical. In addition, most feed intervention projects are not well linked to household nutrition and social needs. An increase in men's earnings from livestock‐related activities may not necessarily translate to improved household nutrition, whereas women tend to prioritize household well‐being. Identifying these gaps will provide ideas for specific measures in project design to guarantee women's participation.

### 3.14 Strategic interventions for improving feed resources in West African Sahel

#### 3.14.1 Identify site‐specific feed intervention

Developing good and sustainable options to address the problem of feed shortage, firstly, it is necessary to assess the existing and potential feed resources, uses, costs, and problems with respect to ruminant production. These evaluations will guide the development of effective strategies to improve nutrition and livestock productivity based on locally available feed resources. Decision‐support tools have the potential to play an important role. For example, FEAST (Feed Assessment Tool, https://ilri.org/feast) is an example of a decision support tool that presents an evolving methodology for conducting rapid appraisals of livestock feed resources in smallholder livestock systems. FEAST uses a systematic method for assessing local feed resource availability and use with a view to design intervention strategies aimed at optimizing feed utilization and animal production. The results of some FEAST approaches conducted in the West African Sahel through an inventory of feed resources in the study areas and identification of priority feed intervention strategies have been documented (Amole & Ayantunde, 2016a, 2016b).

#### 3.14.2 Strengthening fodder market

Strengthening the fodder market structure is very important because fodder markets exist and keep growing in rural areas and cities in the Sahel ranging, from selling of residues at farm gate to selling of mixed concentrates at designated places (Grings et al., 2010). Anandan et al. (2017) highlighted the fact that crop residues are a tradable commodity and have a value chain requiring collection from the field, transport by middlemen, and trading by wholesalers and retailers. Yet the market is still far from being well organized and the value chain is still weak with many unprofessional actors. Therefore, institutional support is needed to organize actors in the forage value chain, establish mechanisms for information sharing, monitor and analyze feed prices, and strengthen stakeholders' knowledge of forage processing and conservation (The World Bank, 2012; Traore, 2016).

#### 3.14.3 Strengthening policy framework

The feed resource sector is largely neglected by policymakers and suffers from limited availability of data and statistics, despite its significant contribution to livestock productivity. Despite surplus production in 2021, major deficits in the availability of maize and soybean feed have occurred in Burkina Faso because of large‐scale exports of these feeds to Nigeria and Niger for higher prices. This lack of a proper framework is one of the main reasons why scaling up of livestock feed interventions fails or does not happen at the expected scale. Effective and coherent feed policies are crucial to capitalize on the growing opportunities offered to smallholder livestock producers by the increasing demand for animal‐source food in the region and low‐ and middle‐income countries in general. Consequently, livestock‐related policies should not be designed in isolation, rather they should be developed through a participatory process that involves all major stakeholders in the livestock and livestock feed value chains, including resource‐poor male and female smallholders and large commercial enterprises.

One effective tool for influencing policy is the formation of multi‐stakeholder innovation platforms (IPs) at local, regional, and national levels. Through an iterative process of consultation, IPs provide an opportunity for main actors in the feed value chain to analyze, plan, and adopt production‐improving measures and influence creation and enactment of enabling policies. While engaging with policymakers at local and national levels to increase understanding of livestock‐related issues, IPs can also be instrumental in identifying shortcomings of existing policies and in formulating new ones. The LSIL has found them to be indispensable for identifying research priorities, ensuring stakeholder's buying in for research programs, receiving advice and critique on ongoing research, and creating enabling policies (Feed the Future innovation Lab for Livestock Systems, 2021).

#### 3.14.4 Strengthening livestock–feed extension

Strengthening extension services by the governmental institutions and nongovernmental organizations is important for better feed production and efficient use of feed resources in West African Sahel. Adoption of better feeding systems and technologies and building knowledge base of the smallholder farmers in feed and feeding systems are strongly influenced by the extension services as reported by Ayantunde et al. (2020). In a study by the authors on determinants of sustainable intensification practices in mixed crop and livestock systems in Burkina Faso, one of the key findings was that access to extension services is an important determinant of adoption of intensification practices in the study sites, which reaffirmed the vital role of extension services in adoption of agricultural technologies. Partnership with the extension services in the co‐design, demonstration, and evaluation of the feed technologies is also important for enhancing adoption of developed technologies. For example, in southern Mali, an improved feed trough for small ruminants was co‐designed and co‐evaluated by research institute and the local nongovernmental organization, and this facilitated strong adoption of the technology by the smallholder farmers (Ayantunde et al., 2020).

#### 3.14.5 Differentiated management and feeding of different ruminant species

Given the multipurpose nature of livestock farming in the Sahel, farmers traditionally practice differentiated management such as, for example, supplementing ready‐to‐be‐sold and lactating animals with more nutritious feed than dry and unproductive animals. Small ruminants, being capable of more effective consumption of nutritious foliage from browse‐dominated rangelands, are usually not supplemented, while larger animals such as lactating cows are given supplementary feeding, which include concentrates, hay, crop residues etc. Differentiated management of different ruminant species in West African Sahel has been reported by some authors (Bayala et al., 2014; Fernandez‐Rivera et al., 2005; Zougmore et al., 2016). Supplements are normally fed to the productive animals particularly pregnant and lactating cows, sheep, and goats; animals being fattened for market, and young animals for their growth. Nonproductive animals are often allowed to lose weight in the dry season, which they regain in the wet season when feed resources, particularly natural pastures are normally abundant. Species differentiation in the Sahel can be by gender and ethnic groups. Women generally prefer small ruminants while the pastoral ethnic groups like Peulh generally prefer cattle as this is an indicator of their wealth status. Species differentiation in West African Sahel has also been driven in the past four decades by droughts, which has favored rearing of small ruminants more than cattle as an adaptation strategy to climate change (Zougmore et al., 2016). Consequently, the population of small ruminants has increased significantly in the Sahel and this trend is likely to continue with climate change.

## 4 CONCLUSION

This review shows that there is a wide diversity of feed types across different agroecological zones in the Sahel that vary tremendously in quality, yield, and seasonality. Among these feed resources that include naturally occurring pastures, crop residues, agro‐industrial by‐products, and household by‐products), crop residues constitute the second most important source of livestock feed after pastures. Seasonal variation in availability and quality of particularly pastures, and the low quality of crop residues result in low livestock productivity in the region. As crop production intensifies and expands with improved seed systems and good management practices, there will be an increased availability of crop residues in the Sahelian zone. To meet the rapidly increasing demand for consumption of animal‐source food, it will be important to harness and leverage the increased crop residue production by coupling it with an improvement of the nutritive value to optimize livestock productivity. Feed inventories need to be developed for each Sahelian country, to catalogue what is available and plan for crisis like drought and conflicts, which are already pervasive in the region. Solutions include promoting and enabling wide‐ scale adoption of improved forage varieties and species with higher yield and nutritive value, as well as adoption of proven methods for forage preservation and crop residue quality improvement. Furthermore, strengthening feed markets and marketing is critical, particularly strengthening the weak links among feed value chain actors that limits feed marketing. Public and private investments are necessary for the dissemination of information and skills through trainings, investment in appropriate technology and machinery, feed storage and transport strategies to improve the production, marketing, and use of livestock feed in the Sahel.

## AUTHOR CONTRIBUTIONS

Tunde Amole: Conceptualization; Data curation; Formal analysis; Methodology; Resources; Writing‐original draft; Writing‐review & editing. Ayantunde Augustine: Conceptualization; Data curation; Formal analysis; Funding acquisition; Investigation; Resources; Validation; Writing‐original draft; Writing‐review & editing. Mulubrhan Balehegn: Data curation; Formal analysis; Project administration; Resources; Supervision; Writing‐original draft; Writing‐review & editing. Adegbola T. Adesogoan: Conceptualization; Data curation; Formal analysis; Funding acquisition; Investigation; Project administration; Resources; Supervision; Validation; Writing‐review & editing.

## CONFLICT OF INTEREST

The authors declare that there is no conflict of interest.
